# Outcomes of Acute Gallstone Disease During the COVID-19 Pandemic: Lessons Learnt

**DOI:** 10.7759/cureus.26198

**Published:** 2022-06-22

**Authors:** Maitreyi S Patel, Joel J Thomas, Xavier Aguayo, Dita Chaloupkova, Princely Sivapregasm, Vivian Uba, Sayed Haschmat Sarwary

**Affiliations:** 1 General Surgery, Barking, Havering and Redbridge University Hospitals NHS Trust, Romford, GBR; 2 Surgery, Barking, Havering and Redbridge University Hospitals NHS Trust, Romford, GBR

**Keywords:** acute pancreatitis, waiting list, outcomes, recurrence, covid-19, gallstone disease, acute cholecystitis

## Abstract

Introduction

This study aims to compare the patient demographics and management of acute manifestations of gallstone disease during the COVID-19 pandemic with an equivalent period in 2019 and assess the differences in recurrence patterns throughout the first and second waves of the pandemic in the UK.

Methods

A retrospective cohort study of all adult patients aged >16 years presenting to the emergency department at a large District General Hospital with symptoms related to gallstones. Data were obtained from electronic patient records. The primary outcomes were incidence and management of gallstone disease, while secondary outcomes studied included length of stay, readmission rate, and recurrence. Data were tabulated and analyzed using Excel (Microsoft, 2016 version). Chi-square and t-test were used as appropriate. One way ANOVA test was used to compare data of three groups.

Results

Fifty-one patients presented during the period of first-wave and 105 patients during the second wave as compared to 71 patients in the study period in 2019. The median age of patients during the first wave of COVID was significantly higher than pre-COVID in the second wave. During both the waves of the pandemic, there was no significant difference in patients presenting with cholecystitis compared with 2019 (47 and 94 in the first and second wave, respectively, versus 60 in 2019; p-value 0.39). There was no significant increase in the use of cholecystostomy, and the use of radiological investigations was comparable. There was no significant difference in recurrence and readmissions. The majority of the patients still await surgery.

Conclusion

During the pandemic, older patients with higher co-morbidity presented with acute gallstone disease. Conservative management was effective in the management of these patients.

## Introduction

The COVID-19 pandemic has resulted in a worldwide reorganization of healthcare services. During the first wave of the pandemic, there was a halt in elective non-cancer surgery. Multiple studies have shown a decline in the number of surgeries performed during the COVID-19 pandemic; however, there have been different experiences in terms of case severity [1-4]. As the pandemic progressed, the understanding of COVID-19 improved, and new pathways were organized to manage surgical patients.

Gallstones present one of the common emergency surgical problems. 15% of adults in the UK suffer from gallstone disease, and 20% of these are symptomatic [5]. Approximately 1-3% have an annual risk of developing complications, including acute pancreatitis, acute cholecystitis, and cholangitis, which could result in frequent hospitalization [6]. Laparoscopic cholecystectomy is the gold standard procedure for the management of symptomatic gallstones. The national guidance from NICE recommends that cholecystectomy be performed within the first week of diagnosis of acute cholecystitis and the index admission for pancreatitis [5]. Untreated symptomatic gallstones may result in recurrence of symptoms and readmissions. During the pandemic, Intercollegiate Guidelines advised non-surgical management for acute biliary disease or the use of cholecystostomy drains [7]. As a result, there was a decline in the number of cholecystectomies performed during the pandemic. This was reflected in the report by the MEGAVID (ManagEment of GAllstone disease during the COVID-19 pandemic) clinical investigator group, which reported a 72.2% decrease in the rate of cholecystectomy since the onset of the pandemic [1]. Consequently, there was an increased risk of readmissions and complications arising from the untreated gallstone disease.

Our hospital is a large Acute District General Hospital serving a diverse population of different ethnicities. We have a dedicated upper GI surgery unit at our hospital. During the pandemic, surgical services were reorganized to maintain an adequate therapeutic path, especially for surgical emergencies. Adopting a conservative approach to managing gallstone disease is more likely to recur symptoms and complications due to the unaddressed underlying pathology, i.e., gallstones. In order to assess this, we conducted a retrospective study to compare the patient demographics and management of acute manifestations of gallstone disease during the COVID-19 pandemic with an equivalent period in 2019 and assess the differences in recurrence patterns throughout the first and second wave of the pandemic in the United Kingdom.

This study was presented as a talking poster at the Association of Surgeons of Great Britain and Ireland (ASGBI) conference 2022.

## Materials and methods

This was a retrospective cohort study of all adult patients aged >16 years presenting to the emergency department with symptoms related to gallstones, including biliary colic, acute cholecystitis, gallstone pancreatitis, and choledocholithiasis. The study was approved by the divisional governance committee and complied with all local and institutional guidelines on information and research governance. Data were obtained from the hospital data information department. This study has been reported in line with the 'Strengthening the reporting of cohort studies in surgery' (STROCSS) criteria [8]. This study is registered with Research Registry with the unique identifying number (UIN) research registry 7785 [9].

Our hospital is a large Acute District General NHS Hospital in England, providing elective and emergency surgical services to a population of approximately 750,000 from a wide range of social and ethnic groups across North East London. Following the announcement of the pandemic, our hospital made sustained efforts to ensure the safety of patients and staff. There was a reorganization of the surgical services in the hospital. Pathways were implemented, including RT-PCR testing of all patients at the time of admission, and isolation as per the test result, while simultaneously beginning appropriate treatment. All surgeries were performed using personal protective equipment following National guidance. During the first wave, emergency cholecystectomy was reserved only for complications. We studied the differences in the demographics and management of gallstone disease during the first wave of the COVID-19 pandemic (March 2020 to May 2020) and the second wave (October 2020 to February 2021). We compared the results with a similar period in 2019 (March 2019 to May 2019). Anonymized patient data were retrospectively collected using electronic patient records. Patient demographics included age and gender. Charlson Comorbidity Index (CCI) was adopted to evaluate the surgical risk [10].

The duration of symptoms, and laboratory results, including white cell count, C-reactive protein levels, liver function test, and renal function, were recorded. Radiological investigations, COVID status, treatment received, hospital stay length, symptoms recurrence, and 30-day readmission rate were recorded. The diagnosis of patients on representation was reviewed. Data from hospital electronic patient records for six months post first presentation was reviewed to assess readmission more than 30 days after the first presentation. The waiting time between the onset of symptoms and surgery was recorded. Patients with acute cholecystitis were stratified using the 2018 Tokyo Guidelines for grading the severity of acute cholecystitis [11].

The primary outcome studied was the incidence and management of gallstone disease. Secondary outcomes studied included length of stay (LOS), defined as the period between admission and discharge in days, and readmission rate, defined as readmission that occurred due to the diagnosis of gallstone disease or its complication and recurrence, which was considered as any visit to the ED (including patients with and without hospitalization) due to relapse of symptomatic gallstone related to disease after discharge. We also assessed the waiting time, defined as the period between a patient being added to the waiting list and the date of surgery. Data were tabulated and analyzed using Excel (Microsoft, 2016 version). Continuous variables were summarized using the median. Descriptive statistics were reported as means with standard deviations for continuous variables and as numbers and percentages for categorical variables. Statistical analysis was performed using a t-test for continuous variables and a chi-square test for categorical variables. The One-way ANOVA test was employed to compare parametric data between three independent groups. The Bonferroni test was used in the postHoc analysis. A p-value of<0.05 was considered statistically significant.

## Results

Fifty-one patients presented during the first wave and 105 patients during the second wave as compared to 71 patients in the study period in 2019. The majority of the patients were females. The median age of patients during the first wave of COVID was significantly higher than pre-COVID in the second wave (Table 1).

**Table 1:** Patient demographics *ANOVA   #Chi square test; NR-Not recorded; CCI- Charlson Comorbidity Index

	Pre-COVID	First wave	Second wave	p-value
N	71	51	105	
Male	17 (24%)	24 (47%)	41 (39%)	0.02#
Female	54(76%)	27(53%)	64(61%)	
Median Age (Range)	51(25-92)	59(26-95)	48 (16-99)	0.046*
CCI mean	2.53	3.25	2.12	0.01*
CCI	68 (96%)	43(84%)	98(93%)	
CCI>5	3(4%)	8(16%)	7 (7%)	
BMI NR	25	28	42	
Mean BMI	29.58	28.68	30.82	

There was no significant difference in blood results in terms of inflammatory markers and liver function tests of patients during the period.

As depicted in Table 2, during both the waves of the pandemic, there was no significant difference in patients presenting with cholecystitis compared with 2019 (24 and 47 versus 38; p-value 0.24). The number of patients presenting with milder forms of the disease like biliary colic was lower during the pandemic. Though the number of patients presenting with gallstone pancreatitis increased during the second wave, the difference was insignificant. As expected, there was a decrease in the number of cholecystectomies performed during the pandemic. Interestingly, there was no significant increase in the number of cholecystostomies performed (Table 2). One patient underwent emergency surgery during the first wave of gall bladder perforation, and both patients who underwent emergency surgery during the second wave had cholecystitis with pancreatitis.

**Table 2:** Clinical diagnosis and management *ANOVA   #Chi square test Abx- Antibiotics; ERCP- Endoscopic Retrograde Cholangio Pancreatography; CBD- common bile duct

	Pre-COVID	First wave	Second wave	p-value	
Duration of symptoms	2(1-5)	3(1-20)	3(1-14)	0.32*	
Diagnosis					
Cholecystitis	38 (54%)	24 (47%)	47 (45%)	0.24#	
Cholecystitis Tokyo 1	26 (37%)	17(33%)	36 (34%)		
Cholecystitis Tokyo 2	7 (10%)	7(14%)	11 (10%)		
Cholecystitis Tokyo 3	5 (7%)	0	0		
CBD stones	17 (24%)	13(25%)	24(23%)		
Pancreatitis	12 (17%)	13 (25%)	33 (31%)		
Biliary colic	4 (6%)	1 (2%)	1(1%)		
Admission	54 (76%)	45 (88%)	94 (90%)	0.04#	
Treatment					
Antibiotics	47 (66%)	44 (86%)	80 (76%)	0.39#	
Abx+Cholecystostomy	3 (4%)	4 (8%)	9 (9%)		
Index Cholecystectomy	4 (6%)	1 (2%)	2 (2%)		
ERCP	12 (17%)	12 (24%)	22 (21%)		
Length of Stay	3	3	5	0.01*	

Interestingly, the number of admissions was higher during the pandemic than during the study period in 2019 (p-value 0.04). Patients' median length of stay was significantly higher during the second wave of the pandemic. Two patients were COVID positive during the first wave, while during the second wave, six patients tested positive for COVID.

The number of patients presenting with recurrence of symptoms and readmissions within 30 days of discharge were higher during the first wave, while the readmissions after 30 days post-discharge were higher during the second wave (p-value 0.73 and 0.35); however, this difference was not significant. There was a significant difference between the diagnoses at the time of recurrence over the study periods (p<0.05) (Table 3). One patient re-presented after a cholecystectomy during the pre-COVID period, and three patients during the second wave-all these patients presented with pancreatitis. During the first and second waves of the pandemic, pancreatitis was the most common cause of representation in patients. In contrast, during the pre-COVID period, most patients presented with biliary colic. There were no mortalities due to gallstone disease.

**Table 3:** Outcomes after initial management *One-way ANOVA   #Chi square test; CCI- Charlson Comorbidity Index; CBD- Common bile duct; ERCP- Endoscopic Retrograde Cholangio Pancreatography

Recurrence/readmission	Pre-COVID	First wave	Second wave	p-value
N	19 (26.76%)	17 (33.34%)	32 (30.48%)	0.73#
Age	53	59.5	50	
Male	3 (16%)	8 (47%)	13 (41%)	0.1#
Female	16 (84%)	9 (53%)	19 (59%)	
Duration of symptoms at 1st presentation	2	3	2	
CCI<5	18 (95%)	16 (94%)	31 (97%)	
CCI>5	1 (5%)	1 (6%)	1 (3%)	
Diagnosis on first presentation				
CBD stones	5 (26%)	1(6%)	8 (25%)	0.042#
Pancreatitis	1 (5%)	5 (29%)	12 (38%)	
Cholecystitis	13 (68%)	11 (65%)	12 (38%)	
Treatment on initial presentation				
Antibiotics	17 (89%)	15 (88%)	26 (81%)	0.79#
Abx+Cholecystostomy	1 (5%)	2 (12%)	3 (9%)	
ERCP	4(21%)	4 (24%)	7 (22%)	
Cholecystectomy	1 (5%)	0	3 (9%)	
Diagnosis on recurrence				
Biliary colic	13 (68%)	1 (6%)	7 (22%)	0.00475#
Obstructive jaundice	1 (5%)	4 (24%)	2 (6%)	
Cholecystitis	1 (5%)	4 (24%)	6 (19%)	
Pancreatitis	3 (16%)	7 (41%)	15 (47%)	
Cholangitis	1 (5%)	1 (6%)	2 (6%)	
Readmission within 30 days	7 (10%)	11(22%)	15(14%)	0.35#
Readmission >30days	9(13%)	9 (18%)	27(26%)	
Waiting time(mean, in days)	142	223	88	0.001*

Ten out of 71 have undergone cholecystectomy during the pre-COVID period, while amongst the patients presenting during the first wave, eleven out of 51 have undergone cholecystectomy, and twenty-five amongst the 105 patients during the second wave have had surgery. Amongst the patients who underwent cholecystectomy, the mean time between first admission and cholecystectomy in 2019 was 142 days. In 2020, it was 223 days during the first wave, while in the second wave, it was only 88 days (p-value .001) (Table 3).

## Discussion

This study demonstrates the management of gallstone disease during the COVID-19 pandemic at a large acute District General Hospital. During the pandemic, approximately a 50% reduction in emergency department attendance was reported across England due to the difference in healthcare-seeking patterns [12,13]. We observed a decrease in the number of patients presenting with the diagnosis of gallstone disease during the first wave of 2020. The median age of presentation was higher during the first wave (59 years v/s 51 years in 2019), while during the second wave, patients presented at a relatively younger age (48 years). The CCI of patients presenting during the first wave was higher (Table 1). Various studies have reported a change in healthcare-seeking behavior, partly due to apprehensions associated with COVID-19 [14-17]. During the pandemic, we saw a significant increase in admissions due to acute gallstone disease. However, the number of patients presenting to ED with acute gallstone disease was relatively unchanged between the periods. More patients presenting during the first wave of the pandemic had severe cholecystitis, as evidenced by a higher number of patients with Grade 2 acute cholecystitis than in the second wave. However, there was no significant rise in Grade 2 and 3 cholecystitis during the pandemic concerning the first wave (Table 2). Although there was no significant difference in the duration of symptoms, it is possible that a delay in presentation could have increased in cases of grade 2 cholecystitis during the first wave [18]. We observed a rise in admissions due to choledocholithiasis and pancreatitis, suggesting that the patients with moderate to severe disease continued to seek medical help despite the pandemic. This could also reflect untreated disease during the pandemic, which resulted in patients developing more complex problems like choledocholithiasis and pancreatitis.

Considering that we did not observe a significant increase in case severity of cholecystitis in our study, it is possible that a substantial number of mild cases recovered without requiring a hospital visit. This could reflect a substantial role played by primary care practitioners. There may be a possibility that the patients may have been lost to follow-up or may have presented to other hospitals. However, further studies may be reasonable to investigate the necessity of surgical intervention in mild cholecystitis. As anticipated, there was a decrease in the number of emergency cholecystectomies performed during the pandemic.

A large national population-based study in the UK has demonstrated that early intervention by performing emergency cholecystectomy within three days of admission has better outcomes. They also reported higher rates of severe complications like bile duct injury in patients more than eight days after presentation [19]. Hence conservative management of acute cholecystitis is reasonable if an emergency surgery cannot be performed within 72 hours. Considering the pandemic, the risk of COVID and its complications outweighed the advantage of emergency cholecystectomy. There was an increase in the number of cholecystostomies during the pandemic, although this was not significant (Table 2). Peckham-Cooper and colleagues [20] have reported a 7.2% rate of cholecystostomy placement in a cohort of 864 patients with cholecystitis during the pandemic; however, this data was not compared with historical observations.

The Royal College of Surgeons of England, through the Cholecystectomy Quality Improvement Collaborative (Chole-QuIC) [21], has developed a translatable model for the delivery of emergency cholecystectomy services. They observed that a surgeon-led Quality improvement collaborative approach is efficacious at improving the care for patients requiring emergency cholecystectomy. This project may help reduce the variability in the provision of emergency cholecystectomy across the United Kingdom and help identify the specific factors that can expedite the development of these services. However, COVID-19 presented a unique challenge in addressing the delivery of emergency cholecystectomy services. Interestingly, in our study, all patients treated conservatively recovered completely without surgical intervention. Patients' length of stay was higher during the second wave; this could be attributed to the higher number of patients admitted with pancreatitis and CBD stones, which required additional investigations and procedures like ERCP.

The reported incidence of recurrence of symptoms after an initial episode of cholecystitis in patients discharged home without operation is 14, 19, and 29% at six weeks, 12 weeks, and at one year, respectively. Recurrent symptoms include biliary colic in 70%, biliary tract obstruction in 24%, and pancreatitis in 6% of these patients [22]. After an initial episode, recurrent gallstone pancreatitis occurs in 25-76% of patients [23]. The recurrence rates were higher during the pandemic, although this rise was insignificant. It was highest for the patients with acute cholecystitis, while acute gallstone pancreatitis had the second-highest recurrence rate (Table 3). The readmission rate was higher during the pandemic. The most common cause of readmission was pancreatitis in these patients. A substantial number of patients with acute cholecystitis recovered without recurrence. It could be suggested that patients with mild cholecystitis are at least at risk of complications. Further large multicentre studies to investigate this may help guide in triaging patients for cholecystectomy to address the long list of patients awaiting cholecystectomy. 

The pandemic has played a significant role in modifying healthcare services worldwide [24]. It posed a unique challenge due to the re-deployment of staff and logistic reasons. Hence, it was difficult to establish emergency cholecystectomy services during the pandemic. COVID-19' cold' pathways established at alternate sites could address only the elective cancer surgery waiting lists as these cases began to be performed with favorable outcomes [25]. However, even a year into the pandemic, elective surgery for the benign condition continues its struggle to return to pre-pandemic levels. After the first wave, as elective surgical services were still striving hard to return to pre-pandemic levels, the second wave emerged towards the end of 2020. This resulted in a severe blow to these services as Hospitals were overwhelmed, and the staff was re-deployed. 4.5 million patients were awaiting elective treatment in the NHS in November 2019, with an additional 1.2 million added every three months of the pandemic [26]. With the continued pressure on the healthcare services imposed by COVID-19, we require a definitive solution as to the best approach to address the pending benign elective surgery moving forward efficiently. A clear plan for recovering the benign elective surgical services in the event of subsequent waves cannot be emphasized enough to address the ever-growing backlog of cases expediently and safely.

At our Institute, intensive work has been done to reduce waiting times. With ongoing new patients presenting with acute gallstone disorders, and a large number of patients on the waiting list for laparoscopic cholecystectomy, we continue our endeavor to reduce the numbers. Those patients with recurrence of symptoms and severe disease are being prioritized. This has helped reduce the numbers, as can be observed by reducing the number of patients awaiting surgery throughout the pandemic and decreasing the waiting time for surgery patients who presented during the pandemic. More patients presented complications due to gallstones during the pandemic, which warranted expedited surgery.

There are limitations to this study. We appreciate that this is a retrospective study, and all data included was reliant on the information available on electronic patient records. Additionally, we could include only those patients who attended our Trust with recurrence but could not account for those who may have attended other centers. The number of patients awaiting surgery may have been overestimated as there is a possibility that some patients underwent cholecystectomy at a private clinic or in another trust, which cannot be confirmed. This may introduce an element of bias. Since the cohort was from a single-center, the generalizability of the results may be limited. Despite these limitations, this study demonstrated the management of acute gallstone disease at a busy center during both waves of the COVID-19 pandemic. To the best of our knowledge, this is one of the few studies investigating patient management and outcomes. This study highlighted that antibiotics and selective use of percutaneous drainage could be safely used to manage acute cholecystitis.

## Conclusions

During the pandemic, older patients with higher CCI presented with acute gallstone disease. There was a possible delay in presentation until progression to complication due to gallstones. Conservative management was effective in managing these patients, as reflected by the fact that there was no significant increase in recurrence or readmissions. Planning a safe and efficient pathway to recover benign elective surgical services will help mitigate the effect on waiting lists.
